# Miss Blackwall, M. D.

**Published:** 1849-08

**Authors:** 


*Miss Blackwell, M. D.*—Miss Blackwell, the female graduate of Geneva College, is now pr osecuting her medical studies at Paris, at the *Hopital de Maternite.*

